# Causal associations between body fat accumulation and COVID-19 severity: A Mendelian randomization study

**DOI:** 10.3389/fendo.2022.899625

**Published:** 2022-08-03

**Authors:** Satoshi Yoshiji, Daisuke Tanaka, Hiroto Minamino, Tianyuan Lu, Guillaume Butler-Laporte, Takaaki Murakami, Yoshihito Fujita, J. Brent Richards, Nobuya Inagaki

**Affiliations:** ^1^ Department of Diabetes, Endocrinology and Nutrition, Graduate School of Medicine, Kyoto University, Kyoto, Japan; ^2^ Department of Human Genetics, McGill University, Montréal, QC, Canada; ^3^ Centre for Clinical Epidemiology, Department of Medicine, Lady Davis Institute, Jewish General Hospital, McGill University, Montréal, QC, Canada; ^4^ Kyoto-McGill International Collaborative Program in Genomic Medicine, Graduate School of Medicine, Kyoto University, Kyoto, Japan; ^5^ Japan Society for the Promotion of Science, Tokyo, Japan; ^6^ Quantitative Life Sciences Program, McGill University, Montréal, QC, Canada; ^7^ Department of Epidemiology, Biostatistics and Occupational Health, McGill University, Montréal, QC, Canada; ^8^ Department of Twin Research, King’s College London, London, United Kingdom; ^9^ 5 Prime Sciences, Montréal, QC, Canada

**Keywords:** COVID-19, body fat accumulation, body composition, Mendelian randomization, genetics

## Abstract

Previous studies reported associations between obesity measured by body mass index (BMI) and coronavirus disease 2019 (COVID-19). However, BMI is calculated only with height and weight and cannot distinguish between body fat mass and fat-free mass. Thus, it is not clear if one or both of these measures are mediating the relationship between obesity and COVID-19. Here, we used Mendelian randomization (MR) to compare the independent causal relationships of body fat mass and fat-free mass with COVID-19 severity. We identified single nucleotide polymorphisms associated with body fat mass and fat-free mass in 454,137 and 454,850 individuals of European ancestry from the UK Biobank, respectively. We then performed two-sample MR to ascertain their effects on severe COVID-19 (cases: 4,792; controls: 1,054,664) from the COVID-19 Host Genetics Initiative. We found that an increase in body fat mass by one standard deviation was associated with severe COVID-19 (odds ratio (OR)_body fat mass_ = 1.61, 95% confidence interval [CI]: 1.28–2.04, *P* = 5.51 × 10^-5^; OR_body fat-free mass_ = 1.31, 95% CI: 0.99–1.74, *P* = 5.77 × 10^-2^). Considering that body fat mass and fat-free mass were genetically correlated with each other (*r* = 0.64), we further evaluated independent causal effects of body fat mass and fat-free mass using multivariable MR and revealed that only body fat mass was independently associated with severe COVID-19 (OR_body fat mass_ = 2.91, 95% CI: 1.71–4.96, *P* = 8.85 × 10^-5^ and OR_body fat-free mass_ = 1.02, 95%CI: 0.61–1.67, *P* = 0.945). In summary, this study demonstrates the causal effects of body fat accumulation on COVID-19 severity and indicates that the biological pathways influencing the relationship between COVID-19 and obesity are likely mediated through body fat mass.

## Introduction

More than 500 million individuals have been infected by the coronavirus disease-19 (COVID-19) with 6 millions of deaths worldwide to date (1). The severity of COVID-19 varies considerably among individuals, and identifying modifiable risk factors associated with COVID-19 severity is essential for optimizing public health policies, allocating resources, and assisting clinical decisions.

A major risk factor for COVID-19 appears to be obesity. A community-based cohort study involving 6.9 million individuals in England showed a positive association between body mass index (BMI) and COVID-19 severity (2), which was replicated in other independent observational studies (3–5). However, the key limitation of BMI is that it is a crude proxy of obesity because it is calculated only with height and weight and does not consider body composition (i.e., body fat mass and body fat-free mass) (6). Therefore, direct measures of body composition assessed by dual-energy X-ray absorptiometry or bioelectrical impedance analysis might better elucidate the association of body fat accumulation with COVID-19 outcomes. In this regard, two recent studies utilized the direct measures of body composition to evaluate the effect of obesity on COVID-19 (7, 8). However, individuals with increased body fat mass are also more likely to have increased body fat-free mass because there is a positive correlation between body fat mass and body fat-free mass (9). Thus, we have to specifically study the independent effects of body fat mass and body fat-free mass to disentangle the causal effects of obesity on COVID-19.

Regarding a means of exploring the associations between risk factors and outcomes of the interest, observational studies can evaluate correlations but not causations; in fact, interpreting the results of observational studies as a causal relationship relies on untestable and usually implausible assumptions, including the absence of unmeasured confounders and reverse causation (10). Given these limitations inherent to traditional observational epidemiology studies, Mendelian randomization (MR) has emerged as a way to mitigate against such shortcomings through its use of genetic variants as instrumental variables to infer a causal relationship between exposures and outcomes (11, 12). Using MR, we can estimate the causal effects of genetically predicted levels of adiposity-related exposures on COVID-19 outcomes, in contrast to typical observational studies that evaluate only associations. Because genetic alleles are randomly assigned at conception, which is generally well before the onset of the disease, the risk of reverse causation is substantially decreased. Taking advantage of MR analysis, previous studies evaluated causal associations of anthropometric traits of obesity and some direct measures of body composition, such as body fat percentage (7, 13–15). However, none has taken into account the correlation of body fat and fat-free mass and evaluated the independent causal associations of body fat mass and body fat-free mass with COVID-19 outcomes.

In this study, we conducted a two-sample MR to assess independent causal associations of body fat mass and body fat-free mass with COVID-19 severity outcomes using data from the UK Biobank and the COVID-19 Host Genetics Initiative.

## Methods

### Instrumental variables for body fat mass, body fat-free mass, body fat percentage, and BMI

**Figure 1:**
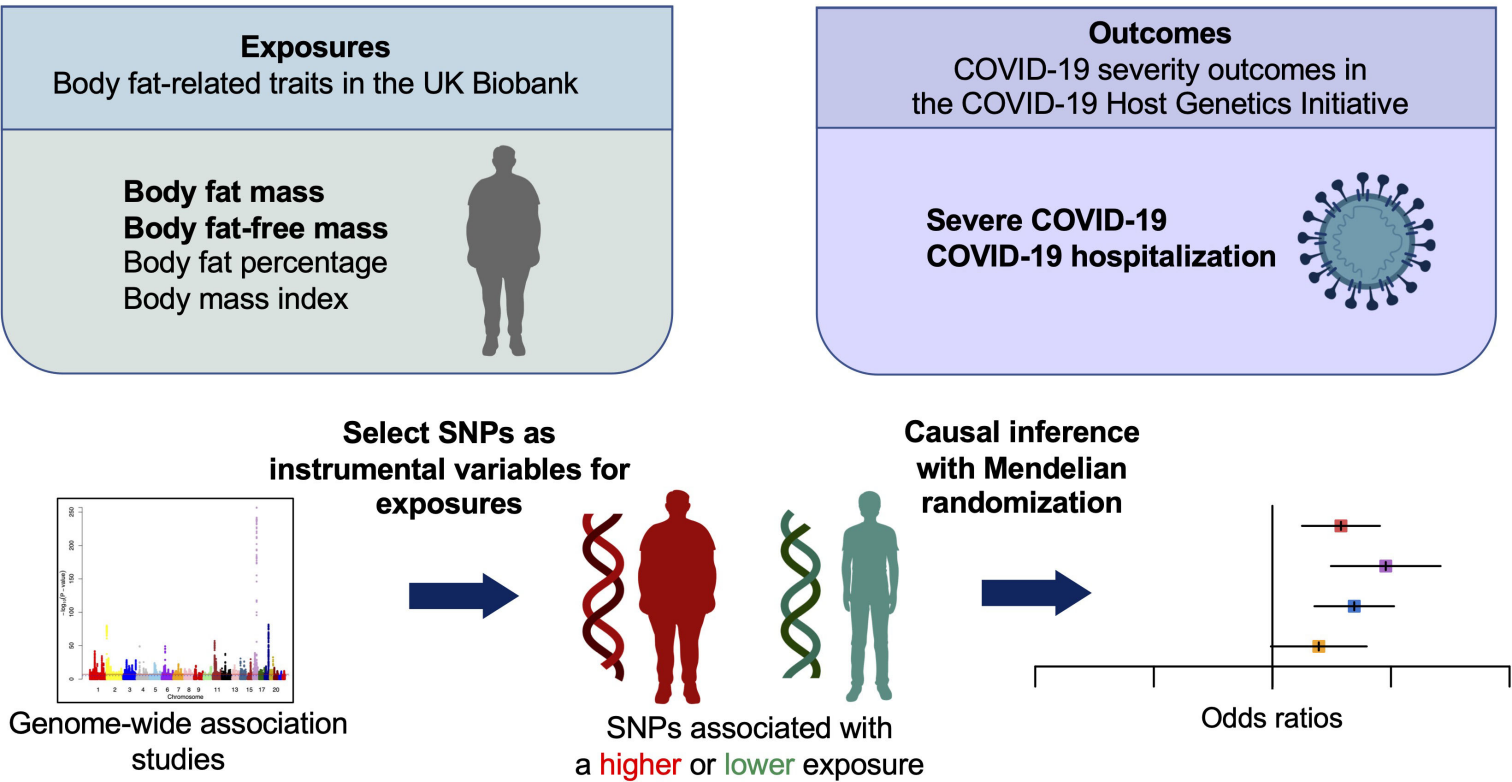
Schematic representation of the Mendelian randomization study. SNPs, single nucleotide polymorphisms.

Instrumental variables were defined as independent genome-wide significant single-nucleotide polymorphisms (SNPs) (*P* < 5 × 10^-8^) for exposure traits. Independence of SNPs was defined as not in linkage disequilibrium with other SNPs (*r^2^* < 0.001 within a 10,000 kilobase [kb] window). The exposures used in this study were body fat mass, body fat-free mass, body fat percentage, and BMI. Body fat percentage and BMI were included as supplementary analyses. To select SNPs used as instrumental variables, we obtained the genome-wide association study (GWAS) results of body fat mass, body fat-free mass, body fat percentage, and BMI from individuals with European ancestry in the UK Biobank (**Figure 1**), using the OpenGWAS and MR-Base platform of the MRC Integrative Epidemiology Unit at the University of Bristol (16). Accession IDs were as follows: body fat mass (ukb-b-19393), body fat-free mass (ukb-b-13354), body fat percentage (ukb-b-8909), and BMI (ukb-b-19953). A full description of the study design, participants and quality control procedures were described in detail previously (17). Briefly, GWAS was performed using 12,370,749 SNPs on 463,005 individuals by BOLT-LMM (18) with the following quality control criteria: Imputation quality (INFO) score > 0.3 for SNPs with a MAF > 3%; INFO score > 0.6 for SNPs with a MAF between 1–3%; INFO score > 0.8 for SNPs with a MAF between 0.5–1%; INFO score > 0.9 for SNPs with a MAF between 0.1–0.5%; SNPs with a MAF below 0.1% were excluded; individuals who were outliers in heterozygosity and missing rates, and individuals with sex-mismatch (i.e. different genetic sex and reported sex) or sex-chromosome aneuploidy were excluded. The fat mass and fat-free mass of the UK Biobank participants were evaluated by performing bioelectrical impedance analysis using the Tanita BC418MA body composition analyzer (Tanita, Tokyo, Japan). We restricted the analyses to individuals of European ancestry to maximize the statistical power, given that the majority of UK Biobank participants were of European ancestry. To select instrumental variables, SNPs were clumped using PLINK (v1.90) according to a linkage disequilibrium threshold of *r^2^* < 0.001 with a clumping window of 10,000 kb using the 1000G European reference panel (16, 19) in order to select an independent SNP with the lowest *P*-value in each linkage disequilibrium block. When a selected SNP was not present in the results of the GWAS of COVID-19 severity outcomes, we instead used a proxy SNP that was in linkage disequilibrium with the selected SNP, with an *r^2^* of ≥0.8 and minor allele frequency of ≤0.3 using 1000G European reference panel as described before (12). We calculated *F*-statistics for the exposure traits and a genetic correlation between body fat mass and body fat-free mass using LDAK (v5.1) (19).

### Severe COVID-19 and COVID-19 hospitalization outcomes

For proxy outcomes of COVID-19 severity, we adopted the outcomes of the COVID-19 Host Genetics Initiative, an international consortium working collaboratively to share data and ideas, recruit patients, and disseminate scientific findings. The outcomes were severe COVID-19 and COVID-19 hospitalization (20). For definitions of COVID-19 outcomes, the severe COVID-19 group was defined as individuals whose death was due to COVID-19, or those requiring hospitalization and respiratory support due to symptoms related to laboratory-confirmed SARS-CoV-2 infection. The COVID-19 hospitalization group was defined as individuals requiring hospitalization due to symptoms associated with laboratory-confirmed severe acute respiratory syndrome coronavirus 2 (SARS-CoV-2) infection. For the definitions of controls in the GWAS data, ancestry-matched controls were sourced from participating population-based cohorts. Controls included individuals whose status of exposure to SARS-CoV-2 was either negative according to electronic health records/questionnaires or unknown (20). We used the largest GWAS summary statistics of the COVID-19 Host Genetics Initiative for severe COVID-19 and COVID-19 hospitalization outcomes in individuals of European-ancestry, excluding those from the UK Biobank. The datasets corresponding to each outcome were as follows: severe COVID-19 (cases: 4,792; controls: 1,054,664; dataset ID: COVID19_HGI_A2_ALL_eur_leave_ukbb_23andme_20210107 from data release 5) and COVID-19 hospitalization (cases: 14,652; controls: 1,114,836; and dataset ID: COVID19_HGI_B2_ALL_eur_leave_ukbb_23andme_20210622 from data release 6). We note that the COVID-19 Host Genetics Initiative’s data release 6 did not include ancestry-specific GWAS for the severe COVID-19 outcome and also that the latest data release 7 did not include GWAS in European-ancestry individuals excluding those from the UK biobank. Hence, we used data release 5 for the severe COVID-19 outcome and data release 6 for the COVID-19 hospitalization outcome to minimize bias due to sample overlap or genetic confounding due to population stratification.

### Mendelian randomization

We performed univariable MR using the inverse variance weighted method (hereinafter referred to as univariable MR) to evaluate the relationship of body fat mass, body fat-free mass, body fat percentage, and BMI with severe COVID-19 and COVID-19 hospitalization. Univariable MR is a weighted linear regression model in which the effect of genetic variants *i* (*i* = 1 … *n*) on an outcome ${\hat{\beta }}_{Y_{i}}$ is regressed on the effect of the same genetic variant *i* on the exposure ${\hat{\beta }}_{X_{i}}$ weighted by the inverse of the squared standard error $\left(se{\left({\hat{\beta }}_{Y_{i}}\right)}^{-2}\right)$ . The estimated total effect ( *θ* ) of the exposure on the outcome can be formulated as follows:


$$\begin{array}{cc} {\hat{\beta }}_{Y_{i}}=\;\theta {\hat{\beta }}_{X_{i}}+\;\varepsilon_{i}, & \varepsilon_{i}\;\sim \;N\left(0,\;se{\left({\hat{\beta }}_{Y_{i}}\right)}^{-2}\right) \end{array}$$

**Figure 2:**
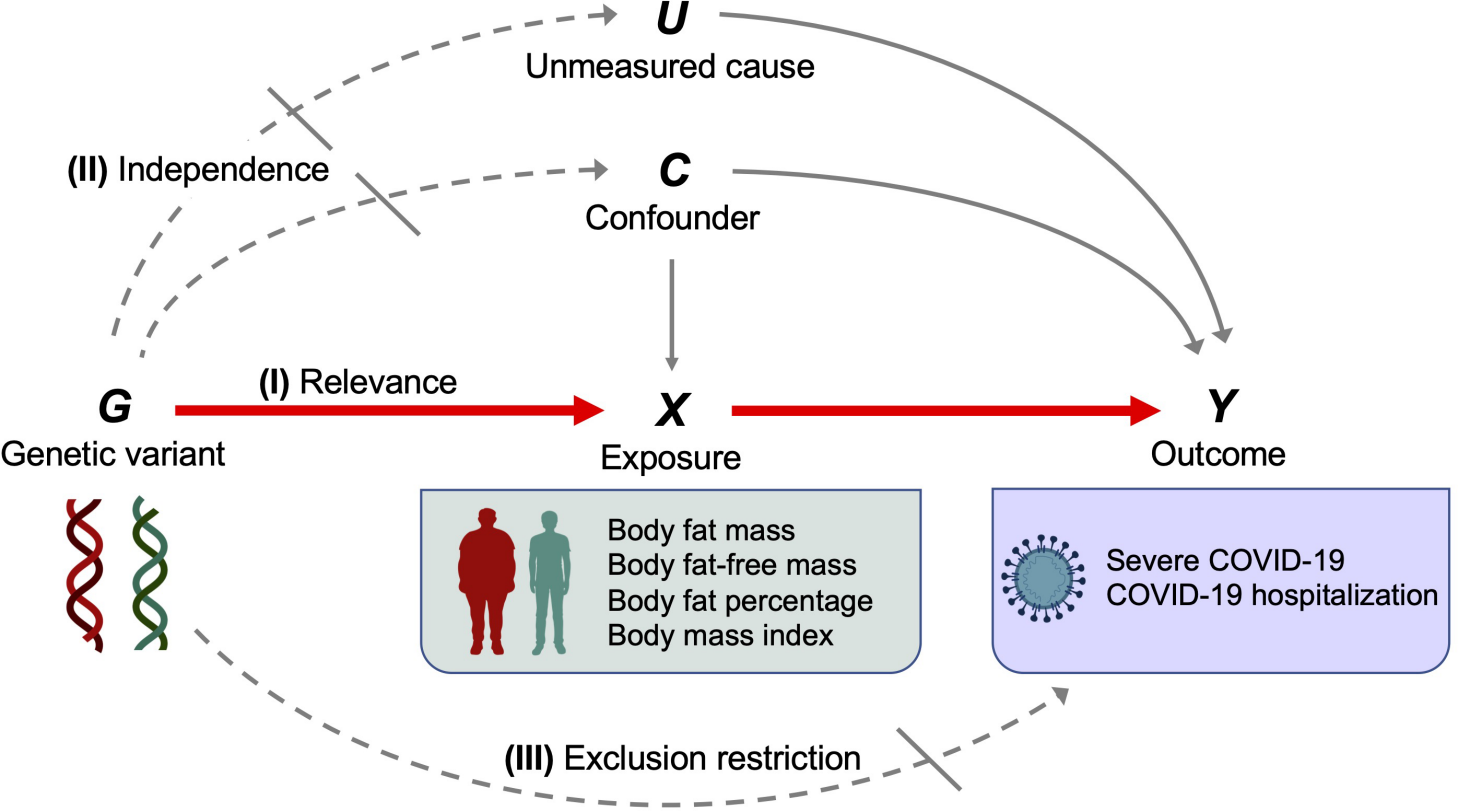
Canonical diagram illustrating the instrumental variable assumptions made in the Mendelian randomization analyses. Genetic variant *G* is used as an instrumental variable for exposure *X* (body fat mass, body fat–free mass, body fat percentage, or body mass index) to evaluate the causal effect of *X* on the outcome *Y* (severe COVID–19 or COVID–19 hospitalization). Instrumental variable assumptions include the following: (I) Relevance–genetic variant *G* is associated with exposure *X*. (II) independence–genetic variant *G* does not share the unmeasured cause or the confounder with the outcome *Y*. (III) exclusion restriction–genetic variant *G* does not influence the outcome *Y* except through the exposure *X*. Red solid arrows represent causal effects, gray solid arrows represent causal effects of the unmeasured cause or confounder that do not violate the instrumental variable assumptions, dashed arrows represent causal effects that are specifically prohibited by the instrumental variable assumptions.

The instrumental variable assumptions are as follows: (I) Relevance–genetic variant is associated with the exposure. (II) independence–genetic variant does not share the unmeasured cause or confounder with the outcome. (III) exclusion restriction–genetic variant does not influence the outcome except through the exposure (11, 12). These assumptions are illustrated by a canonical diagram in **Figure 2**.

Multivariable MR was performed using the inverse variance weighted method (hereinafter referred to as multivariable MR). This is an extension of univariable MR, in which the effects of genetic variant *i* (*i* = 1 … *n*) on the outcome $\left({\hat{\beta }}_{Y_{i}}\right)$ are regressed on the effect of genetic variant *i* on two exposures of *X_1_* (fat mass) and *X_2_* (fat-free mass). In multivariable MR, genetic variants used as instrumental variables are associated with one or both of the exposures (21).

The causal associations were evaluated using odds ratios (ORs), which are expressed according to a standard deviation (SD) increase in genetically predicted body fat mass (kg), or body fat-free mass (kg), body fat percentage (%), and BMI (kg/m^2^).

Results with a *P* < 0.0125 were considered statistically significant (*P* = 0.05/4; Bonferroni-corrected significance threshold according to the number of exposures). We note that such a correction is likely overly conservative, given that the exposures are non-independent. MR analyses were performed using TwoSampleMR (v0.5.6) in R (v4.02). This study was conducted in accordance with the STROBE-MR guideline (6, 7). STROBE-MR checklist is provided in **Supplementary Material**.

### Sensitivity analysis

We performed the MR-Egger intercept test, Cochran’s Q test, and the MR-PRESSO global test (22, 23) to detect horizontal pleiotropy, which occurs when instrumental variables influence outcomes through pathways independent of the exposure. MR-Egger relaxes the exclusion restriction assumption and is valid under the Instrument Strength Independent of Direct Effect (InSIDE) assumption that associations of the genetic variants with the exposure trait are independent of direct effects of the genetic variants on the outcome. Deviation of the MR-Egger intercept from zero indicates horizontal pleiotropy. The results of Cochran’s Q test were used to evaluate the heterogeneity of genetic variants used as instrumental variables. Results of Cochran’s Q test were presented with *I^2^* index, based on which the heterogeneity of genetic variants was defined categorically with *I^2^* index as low (*I^2^* index ≤ 25%), moderate (25% < I^2^index ≤ 50%), and high (*I^2^* index > 50%). Additionally, we performed the MR-PRESSO global test, which can detect horizontally pleiotropic outlier SNPs. A significant result indicates the presence of pleiotropic outlier SNPs and this method then generates ORs after removing and correcting for these outliers (outlier-corrected ORs). MR-PRESSO can also be used to evaluate the distortion of the causal estimates before and after the removal of pleiotropic outlier SNPs following the MR-PRESSO distortion test. MR-PRESSO requires at least 50% of the genetic variants to be valid instruments with no horizontal pleiotropy and also relies on the InSIDE assumption. We also performed leave-one-out analyses for all exposure-outcome associations, which repeated univariable weighted MR excluding each SNP to assess whether the overall estimate is driven by a single SNP. We also generated scatter plots and funnel plots to inspect for horizontal pleiotropy.

Results with a *P* < 0.05 were considered to indicate the presence of horizontal pleiotropy for the MR-Egger intercept test, Cochran’s Q test, MR-PRESSO global test, and MR-PRESSO distortion test. Sensitivity analyses were performed with TwoSampleMR (v.0.5.6) and MR-PRESSO (v1.0).

### Ethics statements

The UK Biobank and COVID-19 Host Genetics Initiatives obtained ethics approval from the relevant institutional ethics committees. We used publicly available summary statistics of GWAS results of UK Biobank and COVID-19 Host Genetics Initiative and did not use individual-level data.

## Results

### Instrumental variables for the exposure traits

The characteristics of the exposure traits (body fat mass, body fat-free mass, body fat percentage, and BMI) are presented in **Table 1**. The mean ± SD of body fat mass was 24.9 ± 9.6 kg, body fat-free mass was 53.2 ± 11.5 kg, body fat percentage was 31.4 ± 8.5%, and BMI was 27.4 ± 4.8 kg/m^2^ (**Table 1**). For body fat mass, body fat-free mass, body fat percentage, and BMI, 417, 530 377, and 439 independent genome-wide significant SNPs were identified as instrumental variables from the GWAS results of the UK Biobank, respectively. *F*-statistics for these exposure traits were 502.2, 607.4, 496.9, and 507.6, respectively. The SNPs used as instrumental variables are presented in **Supplementary Table 1**.

**Table 1:** Dataset descriptions.

Data source	Dataset details	Phenotype	Sample size of each dataset	Mean ± SD
UK Biobank	• GWAS in individuals of European ancestry. • Body fat and body fat–free mass were measured using bioelectrical impedance analysis.	Body fat mass	454,137	24.9 ± 9.6 kg
		Body fat–free mass	454,850	53.2 ± 11.5 kg
		Body fat percentage	454,633	31.4 ± 8.5%
		Body mass index	461,460	27.4 ± 4.8 kg/m^2^
COVID–19 Host Genetics Initiative	• Meta–analysis of GWAS in individuals of European ancestry excluding those from UK biobank	Severe COVID–19	Cases: 4,792 Controls: 1,054,664	–
		COVID–19 hospitalization	Cases: 14,652 Controls: 1,114,836	–

### Severe COVID-19 outcome

**Figure 3:**
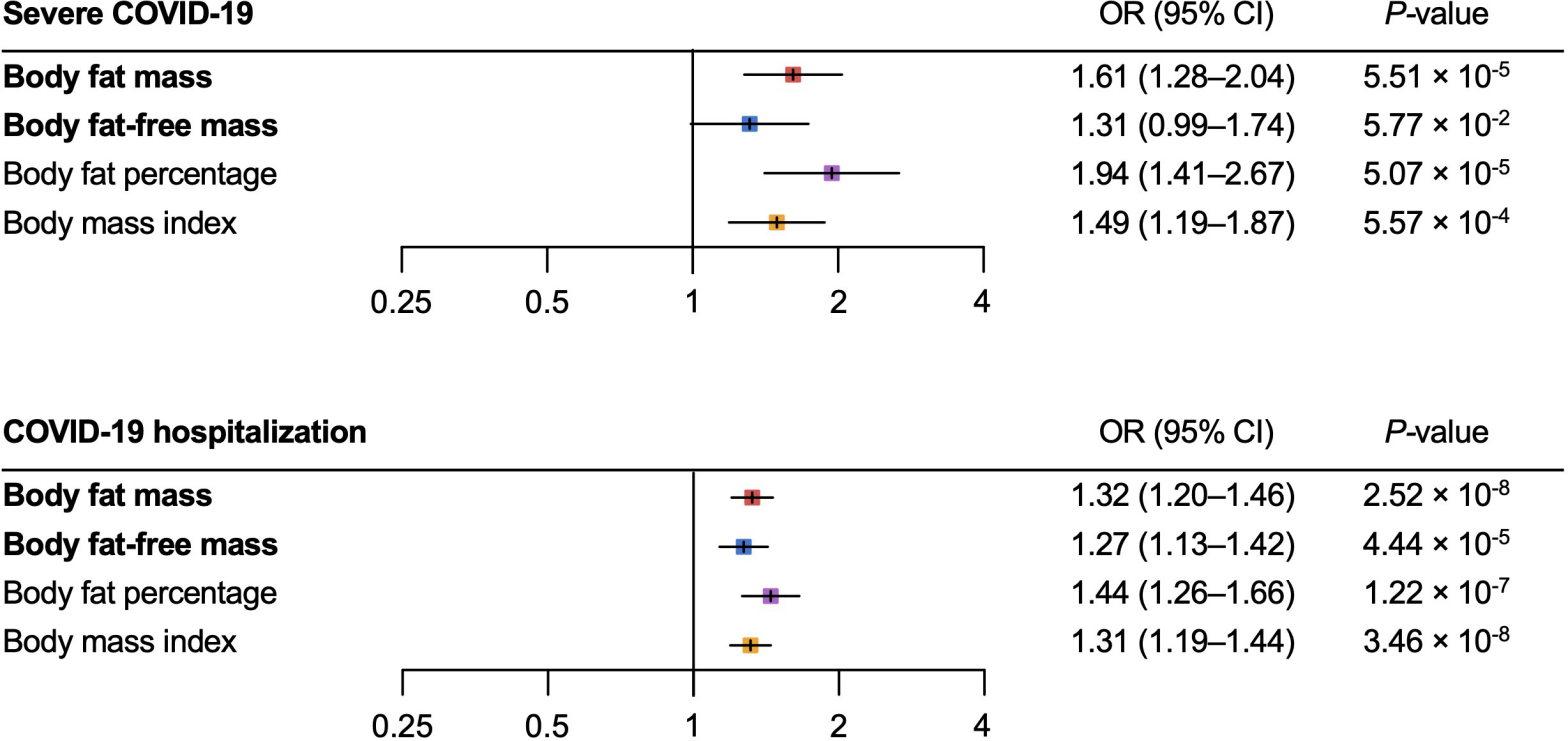
Univariable Mendelian randomization analysis for the severe COVID–19 and COVID–19 hospitalization outcomes. MR, Mendelian randomization.

**Figure 4:**
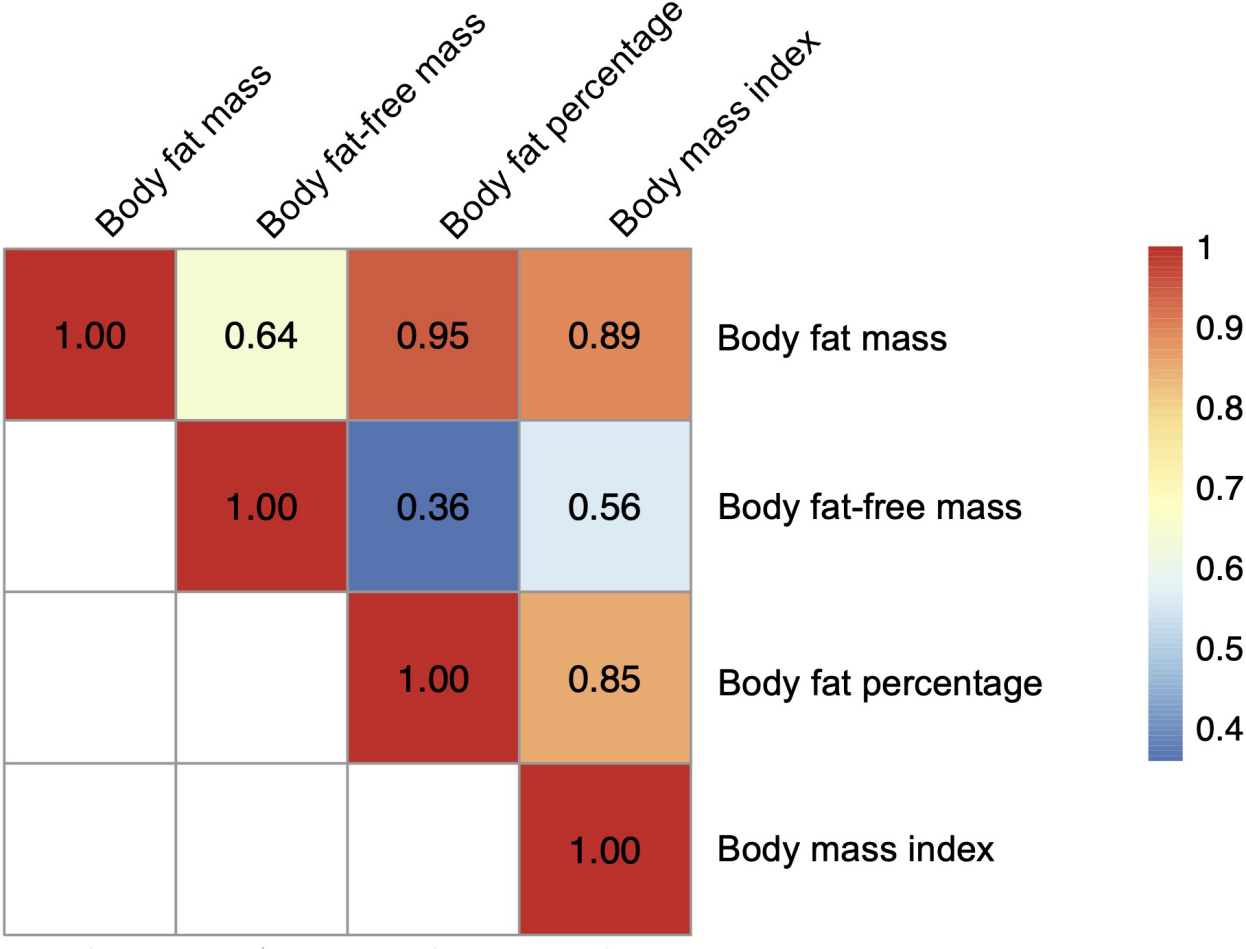
Heatmap for genetic correlation coefficients between the body fat–related traits. Genetic correlations among the four exposures (body fat mass, body fat–free mass, body fat percentage, and body mass index) were analyzed with LDAK using the results of corresponding genome–wide association studies.

**Figure 5:**
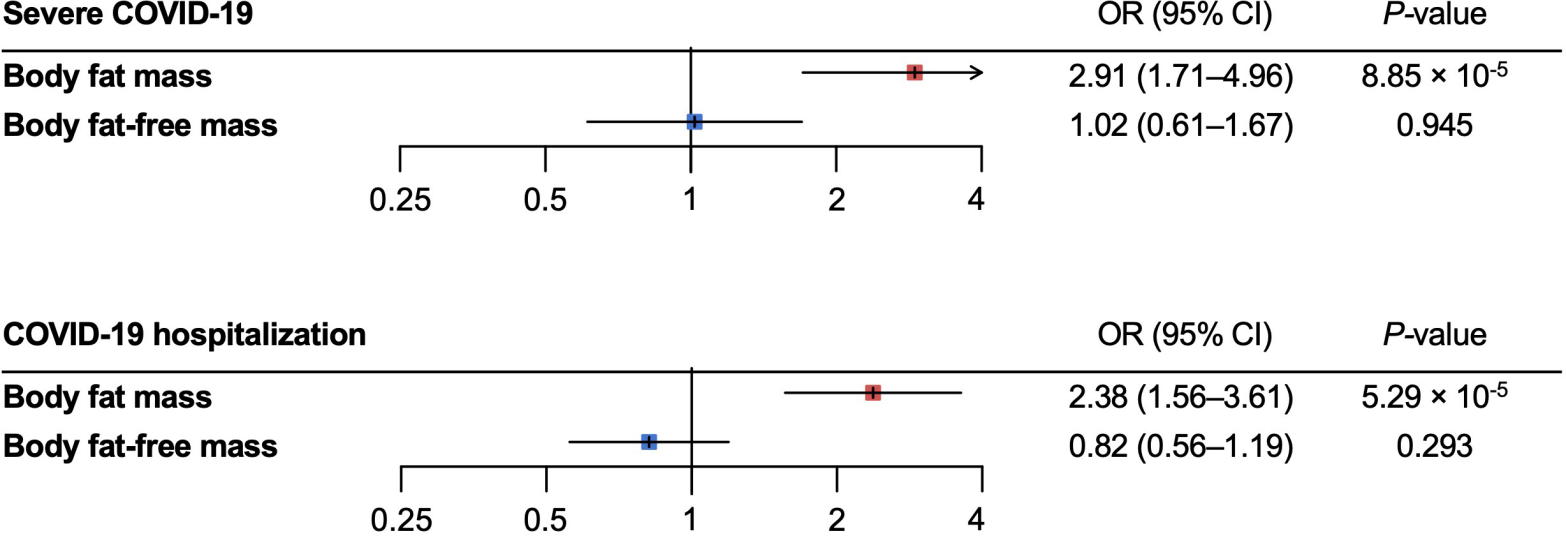
Multivariable Mendelian randomization analysis for the severe COVID–19 and COVID–19 hospitalization outcomes. MR, Mendelian randomization.

For the severe COVID-19 outcome, univariable MR showed that the genetically predicted increase per SD in body fat mass, body fat percentage, and BMI was associated with an increased risk of severe COVID-19 (OR_body fat mass_ = 1.61, 95% CI 1.28–2.04, *P* = 5.51 × 10^-5^; and OR_body fat-free mass_ =1.31, 95% CI: 0.99–1.74, *P* = 5.77 × 10^-2^; OR_body fat percentage_ = 1.94, 95% confidence interval [CI]: 1.41–2.67; *P* = 5.07 × 10^-5^; OR_BMI_ = 1.49, 95% CI: 1.19–1.87, *P *= 5.57 × 10^-4^) (**Figure 3**). Further, as instrumental variables for body fat mass and body fat-free mass were not independent of each other (*r =* 0.64 for the genetic correlation of the two traits) (**Figure 4**), we performed multivariable MR to elucidate the independent causal effects of body fat mass and body fat-free mass on the severe COVID-19 outcome, which showed that only body fat mass was independently associated with the severe COVID 19 outcome (body fat mass: OR_body fat mass_ = 2.91, 95% CI: 1.71–4.96, *P* = 8.85×10^-5^, and OR_body fat-free mass_ = 1.02, 95% CI: 0.61–1.67, *P* = 0.945) (**Figure 5**).

### COVID-19 hospitalization outcome

For the COVID-19 hospitalization outcome, univariable MR showed that a genetically predicted increase per SD in body fat mass, body fat-free mass, body fat percentage, and BMI and was associated with an increased risk of COVID-19 hospitalization (OR_body fat mass_ = 1.32, 95%CI: 1.20–1.46, *P* = 2.52 × 10^-8^; OR_body fat-free mass_ = 1.27 95%CI: 1.13–1.42, *P* = 4.44 × 10^-5^; OR_body fat percentage_ = 1.44, 95%CI: 1.26–1.66, *P* = 1.22 × 10^-7^; OR_BMI_ = 1.31, 95%CI: 1.19–1.44, *P *= 3.46 × 10^-8^) (**Figure 3**). In multivariable MR, only body fat mass was independently associated with COVID-19 hospitalization (OR_body fat mass_ = 2.38, 95%CI: 1.56–3.61, *P* = 5.29×10^-5^; OR_body fat-free mass_ = 0.82, 95%CI: 0.56–1.19, *P* = 0.293), consistent with the findings for severe COVID-19 (**Figure 5**).

### Sensitivity analysis

**Figure 6:**
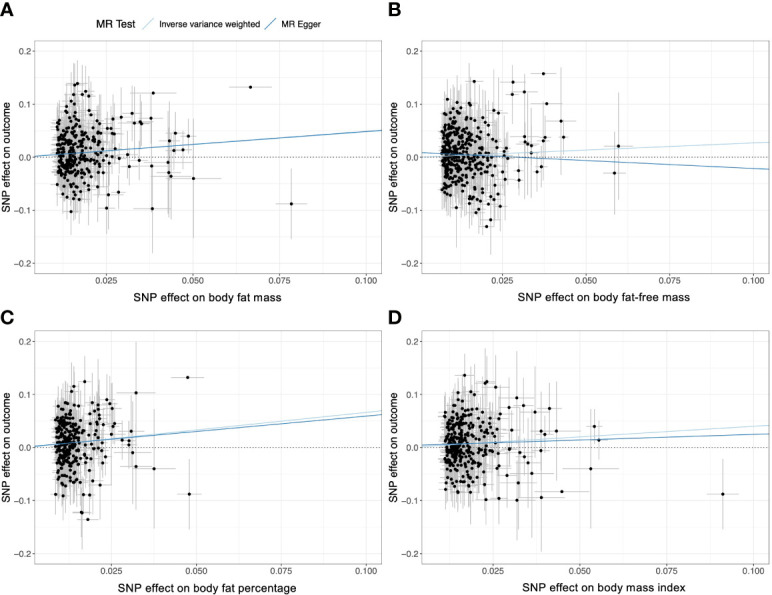
Scatter plots of the univariable weighted MR analyses for **(A)** body fat mass, **(B)** body fat–free mass, **(C)** body fat percentage, and **(D)** body fat mass. Each dot represent a genetic instrumental variable. Two lines represent causal estimate (β_IV_) by the inverse variance weighted method (light blue) and the MR–Egger method (blue). Error bars represent 95%CIs. MR, Mendelian randomization.

We performed MR-Egger, Cochran’s Q test and MR-PRESSO for sensitivity analysis (**Table 2**). In the MR-Egger, the 95%CI results of the MR-Egger intercept (Egger intercept) contained the null hypothesis value zero for all exposure-outcome relationships, suggesting no evidence of horizontal pleiotropy. Heterogeneity estimates of instrumental variables were low according to the *I^2^* index (*I^2^* index were ≤ 25% for all exposure traits). The leave-one-out analyses showed that causal estimates were robust to exclusion of single SNPs (**Supplementary Tables 2**–**5**). Visual inspection of the scatter plots and funnel plots did not suggest biased estimates or pleiotropy (**Figure 6** and **Supplementary Figure 1**). However, MR-PRESSO detected some pleiotropic outlier SNPs in instrumental variables body fat mass, body fat percentage, and BMI with the COVID-19 hospitalization outcome (*P*-value for global test < 0.05). Nevertheless, results with MR-PRESSO after removal and correction for these pleiotropic outlier SNPs were directionally consistent with those from univariable MR, supporting the robustness of the findings with univariable MR. In addition, the MR-PRESSO distortion test detected no significant distortion in the causal estimates before and after the removal of outlier pleiotropic SNPs (**Table 2**).

**Table 2:** Sensitivity analysis results.

Exposures	Outcomes	Sensitivity analysis methods								
		MR–Egger				Cochran’s Q test	MR–PRESSO				
		Egger slope (95% CI)	*P*–value(Egger slope)	Egger intercept (95% CI)	*P*–value(Egger intercept)	*I^2^* index	Global test *P*–value	Outlier–corrected OR (95% CI)	Outlier–corrected *P*–value	Distortion test *P*–value
Body fat mass	Severe COVID–19	1.63 (0.84–3.15)	0.147	–0.0002 (–0.012–0.012)	0.975	4.0	0.273	No outlier	—	—
Body fat–free mass		0.74 (0.38–1.45)	0.378	0.009 (–0.001–0.018)	0.066	5.0	0.551	No outlier	—	—
Body fat percentage		1.79 (0.62–5.14)	0.282	0.001 (–0.013–0.016)	0.874	9.7	0.108	No outlier	—	—
Body mass index		1.24 (0.65–2.37)	0.523	0.004 (–0.008–0.015)	0.542	1.8	0.417	No outlier	—	—
Body fat mass	COVID–19 hospitalization	1.53 (1.17–2.00)	2.31×10^–3^	–0.003 (–0.008–0.002)	0.264	17.3	0.004	1.3351 (1.3348–1.3353)	3.98 × 10^–3^	0.881
Body fat–free mass		1.12 (0.85–1.46)	0.433	0.002 (–0.002–0.006)	0.302	12.3	0.174	No outlier	—	—
Body fat percentage		1.80 (1.17–2.76)	8.04×10^–3^	–0.003 (–0.009–0.003)	0.297	18.4	0.003	1.4498 (1.4493–1.4503)	1.60 × 10^–7^	0.810
Body mass index		1.27 (0.99–1.64)	6.34×10^–2^	0.001 (–0.004–0.005)	0.812	19.5	0.001	1.3085 (1.3082–1.3088)	7.09 × 10^–9^	0.864

## Discussion

In this study, we used two-sample MR to disentangle the independent effects of body fat mass and body fat-free mass and showed that body fat mass, but not body fat-free mass, is independently associated with severe COVID-19 outcomes. First, we performed univariable weighted MR and found that increased body fat mass, along with BMI and body fat percentage, were associated with an increased risk of severe COVID-19 and COVID-19 hospitalization. We further used multivariable MR to disentangle the independent causal effects of body fat mass and body fat-free mass on these outcomes and revealed that only body fat mass was independently associated with the outcomes.

During the COVID-19 pandemic, obesity has emerged as a major risk factor for COVID-19 outcomes. Multiple observational and MR studies suggested that obese individuals present an increased risk of severe diseases, hospitalization, and death due to COVID-19 (2–4, 24). However, observational studies are prone to confounding bias and reverse causation and do not estimate the causal effects of exposures on outcomes. To tackle this problem, recent studies have used MR to estimate the causal effect of obesity on the risk of COVID-19. For instance, the landmark paper from the COVID-19 Host Genetics Initiative showed that BMI was causally associated with an increased risk of COVID-19 hospitalization (20). This was supported by multiple MR studies and our analysis, which included BMI as the supplementary exposure. Other studies also assessed multiple anthropometric traits, including waist circumference, hip circumference, waist-to-hip ratio, and trunk fat ratio as well as BMI to evaluate the effect of adiposity on the risk of COVID-19 (7, 8, 13, 14, 25–31). These MR studies consistently estimated that increases in BMI, waist circumference, and hip circumference are causal for COVID-19 severity (7, 13, 14, 26, 28). On the other hand, the waist–to–hip ratio was not associated with COVID–19 severity (7, 28), contradicting observational studies. These discrepancies may be explained by confounding factors involved in observational studies but also by the limited ability of anthropometric traits to act as proxies for body composition (i.e., body fat mass and fat–free mass). It should also be noted that BMI is a function only of weight and height and an indirect measurement of obesity. Thus, it may not necessarily reflect body composition, which can be directly measured with bioelectrical impedance analysis or dual–energy X–ray absorptiometry (DXA). For example, individuals with similar BMI may have very different body composition, if there are large changes in lean body mass. This highlights the importance of directly measuring adiposity. In this regard, two recent MR studies used GWAS of direct measurements of obesity (i.e., body fat mass, fat–free mass, and body fat percentage) and found that they influence the risk of COVID–19, which was replicated by our univariable MR analyses (7, 8). However, analyses using body composition measurements still have limitations such as the high correlation between body fat mass and body fat–free mass, which was highlighted by our genetic correlation analysis (*r* = 0.64). To the best of our knowledge, the present study is the first to disentangle the independent causal effects of body fat mass and body fat–free mass on COVID–19 severity.

Our multivariable MR showed that one SD increase in body fat mass (9.6 kg) is causally associated with 2.91–fold and 2.38 fold–increase in the risk of severe COVID–19 and COVID–19 hospitalization, respectively, highlighting the burden of body fat accumulation on COVID–19 severity. On the contrary, body fat–free mass were not independently associated with increased risk of severe COVID–19 or hospitalization. We used multivariable MR since most instrumental variables of adiposity affect both fat mass and fat–free mass, although some variants more strongly and proportionally influence fat mass, whereas others influence fat–free mass more strongly. Therefore, multivariable MR can test the differential causal effects of fat mass and fat–free mass. Using this approach, recent MR studies showed differential associations between body fat mass and body fat–free mass with various disorders (9, 32–34). The present findings extend this knowledge to COVID–19. Results from multivariable MR showed that body fat mass but not body fat–free mass was independently associated with severe COVID–19 and COVID–19 hospitalization. The association between body fat mass and COVID–19 severity was strengthened in multivariable MR relative to findings using univariable MR, whereas the effects of body fat–free mass on COVID–19 severity was markedly attenuated in multivariable MR, thereby illustrating the independent causal effects of body fat mass on COVID–19 severity.

The underlying mechanism of these associations remains to be clarified. Obesity is a metabolic disease characterized by systemic changes in metabolism, including insulin resistance, glucose intolerance, dyslipidemia, changes in adipokines (e.g., increased leptin and decreased adiponectin levels), chronic inflammation, and altered immune response, all of which could collectively increase the risk of COVID–19 severity (35–37). In addition, recent studies suggests that adipose tissue is a potential organ for direct infection with SARS–CoV2 in obese individuals (35). The infection of adipose tissue can cause systemic metabolic dysregulation including hyperglycemia, which is known as another risk factor for COVID–19 severity (36). Moreover, obesity causes respiratory dysfunction, including impaired respiratory physiology, increased airway resistance, impaired gas exchange, low lung volume, and low muscle strength, which can also increase the risk of COVID–19 severity. Furthermore, the physical characteristics of obese individuals render intubation and laryngoscopy difficult, which could also aggravate outcomes (37). Further studies are needed to explore the pathways linking adiposity to increased risk of COVID–19 severity.

This study has several strengths. We used an MR design, which minimized bias from reverse causation and confounders, thereby enabling us to test for causal effects, provided compliance with MR assumptions. In this MR study, we used the data from the UK Biobank for the exposure traits (*F*–statistics > 10 for all exposure traits) and COVID–19 Host Genetics Initiative for the outcomes, both of which have large sample sizes, thus increasing the statistical power of the analysis. Furthermore, as proxy measures of body composition, we not only considered BMI, which is a common indirect measure, but also direct measures, including body fat mass, body fat–free mass, and body fat percentage and revealed associations of these traits with COVD–19 severity.

Our study also has important limitations. First, MR analysis relies on several key assumptions, the violation of which compromises causal inference: relevance, independence, and exclusion restriction (**Figure 2**). To test for possible violations of these assumptions, we performed multiple sensitivity analyses. The MR–Egger intercept test did not detect horizontal pleiotropy. Although heterogeneity of effects were detected for certain SNPs when analyzing COVID–19 hospitalization, the removal of outlier SNPs *via* MR–PRESSO still showed results consistent with those from MR inverse variance weighted method. We believe that these sensitivity analyses demonstrate the robustness and validity of the present findings. However, we acknowledge that horizontal pleiotropy is difficult to exclude entirely. Second, regarding exposure traits, we used measures derived from the bioelectrical impedance analysis (i.e., body fat percentage, body fat mass, and body fat–free mass) instead of DXA–derived measures to maximize statistical power. Although the UK Biobank collected DXA–derived measures for body fat mass and body fat–free mass, the sample size was markedly smaller for these measurements (*n* = 5,170). Moreover, although DXA–derived measures are generally more accurate than impedance–derived measures, high correlations between the two were reported for fat mass (*r* = 0.96) and fat–free mass (*r* = 0.86) in the UK Biobank dataset (9). Hence, we believe impedance–derived measures can serve as clinically–relevant exposure traits in the present analysis. Third, we only used summary–level data and did not use individual–level data. Therefore, we could not evaluate the nonlinear relationship between exposures and outcomes. However, it should be noted that MR using summary statistics can still test for the presence of causal effects of exposures on outcomes, even if the exposure–outcome relationship is nonlinear (38). Additionally, a recent prospective cohort study of 6.9 million individuals in the UK suggested that BMI and COVID–19 severity have a linear relationship within a BMI range ≥23 kg/m^2^ (2). Notably, the BMI of a majority of the individuals in the UK Biobank population included in the present analysis fell within this range (≥23 kg/m^2^). Fourth, we restricted our analysis to individuals of European ancestry given that majority of participants in the UK Biobank were of European ancestry. Future studies are warranted to evaluate the generalizability of our findings to other populations. Lastly, we did not evaluate other clinically established risk factors such as diabetes, respiratory, heart, kidney, liver, autoimmune disorders, older age, smoking, and lower socioeconomic status (39). When considering risk factors for COVID–19 severity, we have to take into account phenotypic and genetic correlations. This was highlighted by a recent study showing that the causal effect of diabetes on COVID–19 severity is mediated by BMI (40). Another study also showed that the effect of BMI on severe COVID–19 is partially mediated by socioeconomic status measured by household income (26). Furthermore, obesity is associated with other risk factors for severe COVID–19, including, but not limited to, chronic obstructive lung disease, heart failure, chronic kidney disease, liver cirrhosis, and autoimmune disorders (41–45). The interconnected nature of these risk factors highlights the importance of disentangling the independent causal effect of each risk factor, which requires further investigation.

In summary, the present MR study provides evidence that indicates a causal relationship between body fat accumulation and COVID–19 severity. Because excess fat can be reduced by following an appropriate diet and exercising, it might represent an important modifiable risk factor. Thus, body weight reduction considering direct measurements of body fat (i.e., body fat mass and body fat percentage) can be an effective strategy to reduce the risk of COVID–19 severity.

## Data availability statement

All GWAS summary statistics used in this study are publicly available. The original contributions presented in the study are included in the article/**Supplementary Material**. Further inquiries can be directed to the corresponding authors.

## Ethics statement

Ethical review and approval was not required for the study on human participants in accordance with the local legislation and institutional requirements. The patients/participants provided their written informed consent to participate in this study.

## Author contributions

SY conceptualized and analyzed the data. SY, HM, and JBR wrote the original draft of the manuscript. JBR and NY supervised the study. All authors discussed the results and contributed to the final manuscript.

## Funding

The Richards research group is supported by the Canadian Institutes of Health Research (CIHR: 365825, 409511, 100558, 169303), the McGill Interdisciplinary Initiative in Infection and Immunity (MI4), the Lady Davis Institute of the Jewish General Hospital, the Jewish General Hospital Foundation, the Canadian Foundation for Innovation, the NIH Foundation, Cancer Research UK, Genome Québec, the Public Health Agency of Canada, McGill University, Cancer Research UK [grant umber C18281/A29019] and the Fonds de Recherche Québec Santé (FRQS). JBR is supported by an FRQS Mérite Clinical Research Scholarship. Support from Calcul Québec and Compute Canada is acknowledged. TwinsUK is funded by the Welcome Trust, Medical Research Council, European Union, the National Institute for Health Research (NIHR)–funded BioResource, Clinical Research Facility and Biomedical Research Centre based at Guy’s and St Thomas’ NHS Foundation Trust in partnership with King’s College London. These funding agencies had no role in the design, implementation or interpretation of this study. SY and HM are supported by the Japan Society for the Promotion of Science.

## Conflict of interest

JBR institution has received investigator–initiated grant funding from Eli Lilly, GlaxoSmithKline and Biogen for projects unrelated to this research. He is the founder of 5 Prime Sciences (www.5primesciences.com), which provides research services for biotech, pharma and venture capital companies for projects unrelated to this research. NI received research funds from Terumo Corp., Drawbridge, Inc., and Asken Inc. NI received speaker honoraria from Kowa Co., Ltd., MSD K.K, Astellas Pharma Inc., Novo Nordisk Pharma Ltd., Ono Pharmaceutical Co., Ltd., Nippon Boehringer Ingelheim Co., Ltd., Takeda Pharmaceutical Co., Ltd., Mitsubishi Tanabe Pharma Corp., Sumitomo Dainippon Pharma Co., Ltd., Sanofi K.K., Eli Lilly Japan K.K., received scholarship grant from Kissei Pharmaceutical Co., Ltd., Sanofi K.K., Daiichi–Sankyo Co., Ltd., Mitsubishi Tanabe Pharma Corp., Takeda Pharmaceutical Co., Ltd., Japan Tobacco Inc., Kyowa Kirin Co., Ltd., Sumitomo Dainippon Pharma Co., Ltd., Astellas Pharma Inc., MSD K.K., Ono Pharmaceutical Co., Ltd., Sanwa Kagaku Kenkyusho Co., Ltd., Nippon Boehringer Ingelheim Co., Ltd., Novo Nordisk Pharma Ltd., Novartis Pharma K.K., and Life Scan Japan K.K. NI is an advisory board member of Novo Nordisk. These agencies did not play any role in study design, the collection, analysis, or interpretation of data, the writing of the report, or the decision to submit this paper for publication.

The remaining authors declare that the research was conducted in the absence of any commercial or financial relationships that could be construed as a potential conflict of interest.

## Publisher’s note

All claims expressed in this article are solely those of the authors and do not necessarily represent those of their affiliated organizations, or those of the publisher, the editors and the reviewers. Any product that may be evaluated in this article, or claim that may be made by its manufacturer, is not guaranteed or endorsed by the publisher.

## Supplementary material

Supplementary Figure 1 Funnel plots of the univariable weighted MR for **(A)** body fat mass, **(B)** body fat–free mass, **(C)** body fat percentage, and **(D)** body fat mass. Each dot represent a genetic instrumental variable. Two lines represent causal estimate (β_IV_) by the inverse variance weighted method (light blue) and the MR–Egger method (blue). SE_IV_ represents standard error for each genetic instrumental variable. Error bars represent 95%CIs. MR, Mendelian randomization, IV, genetic instrumental variable.
