# Supplementary figures and images for: Causal associations between body fat accumulation and COVID-19 severity: A Mendelian randomization study
Source: Front Endocrinol (Lausanne). 2022 Aug 3;13:899625. doi: 10.3389/fendo.2022.899625 (PMC9381824; doi:10.3389/fendo.2022.899625)

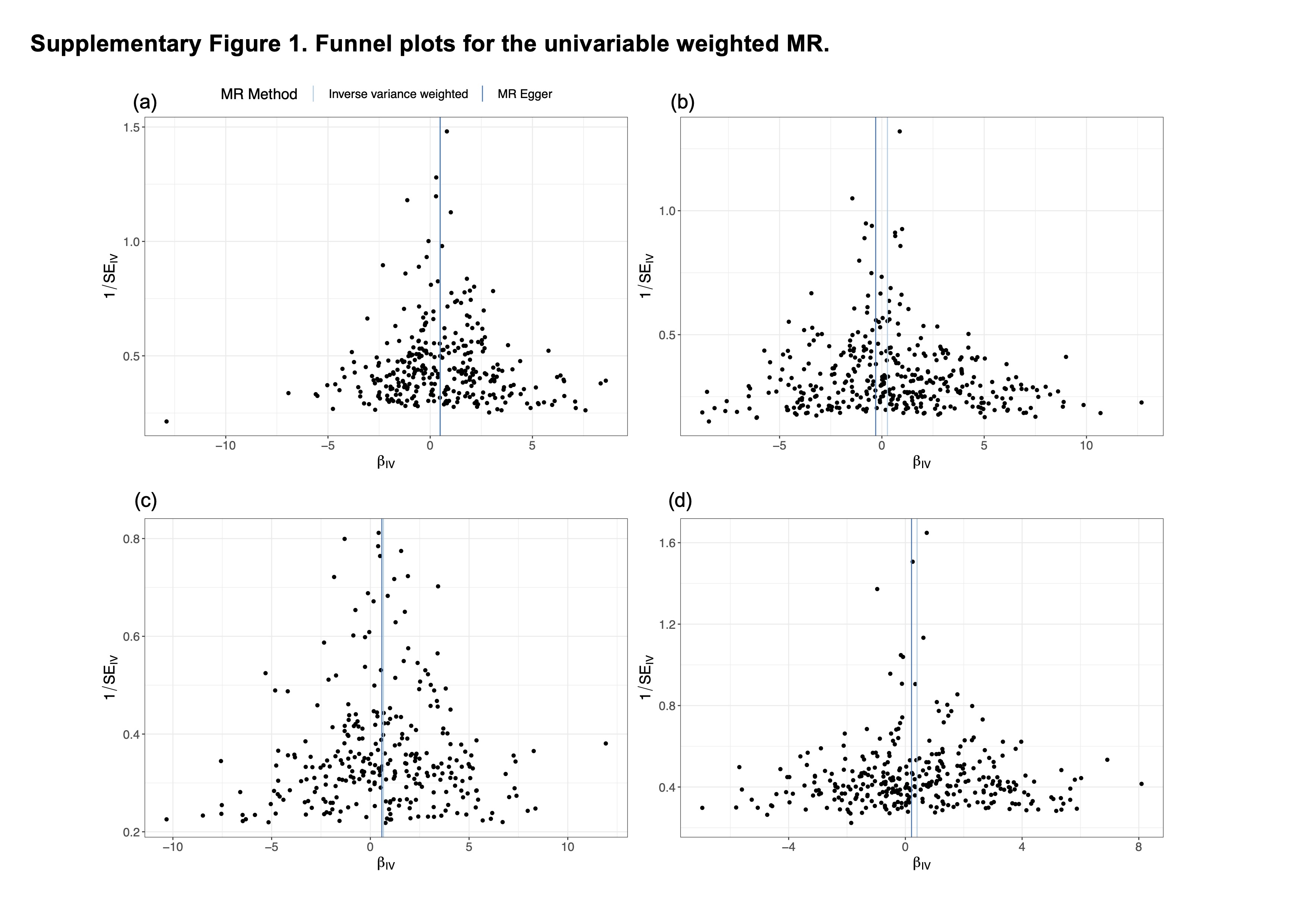

Supplement: Supplementary Figure 1 — Funnel plots of the univariable weighted MR for (A) body fat mass, (B) body fat–free mass, (C) body fat percentage, and (D) body fat mass. Each dot represent a genetic instrumental variable. Two lines represent causal estimate (βIV) by the inverse variance weighted method (light blue) and the MR–Egger method (blue). SEIV represents standard error for each genetic instrumental variable. Error bars represent 95%CIs. MR, Mendelian randomization, IV, genetic instrumental variable. [file Image_1.jpeg]
